# IgA nephropathy pathogenesis and therapy: Review & updates

**DOI:** 10.1097/MD.0000000000031219

**Published:** 2022-12-02

**Authors:** Elmukhtar Habas, Elrazi Ali, Khalifa Farfar, Mahdi Errayes, Jamal Alfitori, Eshrak Habas, Hafedh Ghazouani, Raza Akbar, Fahim Khan, Aisha Al Dab, Abdel-Naser Elzouki

**Affiliations:** a Hamad General Medicine, Doha, Qatar; b Hamad General Hospital, Medicine Department, Doha, Qatar; c Alwakar Hospital, Alwakra, Qatar; d Hamad General Hospital, Doha, Qatar; e Tripoli University; f Quality Department, Hamad Medical Corporation, Doha, Qatar; g Tripoli Children hospital, Tripoli, Libya; h Hamad Medical Corporation, Doha, Qatar; i Hamad General Hospital, Doha, Qatar.

**Keywords:** ARBs, Berger disease, hematuria, IgA nephropathy, proteinuria

## Abstract

IgA nephropathy (IgAN) is the most frequent type of primary glomerulonephritis since the first type was described more than four decades ago. It is the prevalent cause of primary glomerular disease that causes end-stage renal disease. In most patients with IgAN, hematuria is the most common reported symptom, particularly in those with a preceding upper respiratory tract infection. Although the pathogenesis of IgAN is usually multifactorial, autoimmune complex formation and inflammatory processes are the most widely recognized pathogenic mechanisms. Multiple approaches have been trialed as a therapy for IgAN, including tonsillectomy, steroids, other immune-suppressive therapy in different regimens, and kidney transplantation.

**Aim and method:**

PubMed, Google, Google Scholar, Scopus, and EMBASE were searched by the authors using different texts, keywords, and phrases. A non-systemic clinical review is intended to review the available data and clinical updates about the possible mechanism(s) of IgAN pathogenesis and treatments.

**Conclusion:**

IgAN has a heterogeneous pattern worldwide, making it difficult to understand its pathogenesis and treatment. Proteinuria is the best guide to follow up on the IgAN progression and treatment response. Steroids are the cornerstone of IgAN therapy; however, other immune-suppressive and immune-modulative agents are used with a variable response rate. Kidney transplantation is highly advisable for IgAN patients, although the recurrence rate is high. Finally, IgAN management requires collaborative work between patients and their treating physicians for safe long-term outcomes.

## 1. Introduction

Berger and Hinglais first described IgA nephropathy (IgAN) in 1968, and since then, it has been named Berger disease.^[1,2]^ IgAN is typically characterized by prominent immunofluorescent mesangial IgA deposits detected by immunofluorescence microscopy.^[3]^ IgAN is one of the world’s most prevalent glomerulonephritis (GN) diseases.^[4]^ It has been reported that IgAN has a variable prevalence of 5 to 40%. End-stage Renal Disease (ESRD) occurs in around 15 to 20% of IgAN patients within ten years and in over a third (30%–40%) of patients during 20 years of follow-up.^[5]^ IgAN was previously known as a rare and benign cause of recurrent hematuria.^[2]^ A confirmed diagnosis of IgAN is established by renal biopsy, which is not a common practice in some nephrology centers, affecting the accurate assessment of overall disease prevalence. Currently, IgAN is not recognized as a rare or benign condition. The progression of IgAN is linked with the presence of proteinuria (even at lower levels such as (<1 g/d)^[6]^), urine red blood cells (RBC) count, hypertension,^[7]^ elevated serum creatinine, and microscopic lesions (as reported in the Oxford classification) at presentation.^[8]^ These prognostic data may help stratify those patients with the highest need for effective early therapy.

IgAN is less common in black people than in Asian or white populations.^[9]^ IgAN-like kidney injury has been related to autoimmune diseases such as Henoch-Schönlein purpura, chronic hepatitis, systemic lupus erythematosus, dermatitis herpetiformis, and ankylosing spondylitis. IgA glomerular mesangial deposits are frequently detected in histological analyses in all these conditions.

An extensive search in PubMed, Google, Google Scholar, Scopus, and EMBASE was conducted using different texts, keywords, and phrases such as Berger’s disease, the pathogenesis of IgA nephropathy, the clinical presentation of IgA nephropathy, new updates in IgA nephropathy therapy, IgA histology, and hemoptysis with hematuria.

## 2. Presentation

Synpharyngitic nephritis is a synchronous combination of macroscopic hematuria and pharyngitis induced by upper respiratory tract infections, most commonly acute pharyngotonsillitis. IgAN may present with different grades of hematuria (mild, moderate, or severe hematuria). Gross hematuria occurs around the same time as the infection or within the first 2 to 3 days, lasting typically for less than three days and usually manifesting with loin discomfort in about 30% of patients. The loin pain is primarily due to the sudden enlargement of the kidney, leading to stretching of the renal capsule. Gross hematuria predominantly affects children and youth and is usually a self-limiting symptom. Clinically, most patients with IgAN have unimpressive physical findings at the time of initial clinical presentation. However, some patients may acquire hypertension early in the disease. Hypertension prevalence increases as the disease progress to more advanced CKD stages and ESRD. Edema and third-space fluid retention such as pleural effusion and ascites may also be present in nephrotic IgAN patients.

## 3. Causes and associations (Secondary IgAN)

Although most instances of IgAN are idiopathic, an upper respiratory tract infection such as Hemophilus Parainfluenza frequently precedes the disease presentation. IgAN is also associated with other diseases such as liver cirrhosis, HIV infection, Coeliac disease, etc.

### 3.1. Cirrhosis and other liver diseases

IgAN is a relatively prevalent consequence of chronic liver disease, especially alcoholic cirrhosis. Glomerular IgA deposition occurs in more than 30% of patients with cirrhosis and chronic liver disease due to the impaired function of the damaged Kupffer cells, decreasing IgA-containing complexes clearance, and predisposing IgA deposition in the kidneys.^[10]^ Glomerular IgA deposits are prevalent in advanced liver illness, especially in young patients, but most adults have no clinical feature of glomerular disease. After a successful liver transplant, these renal abnormalities commonly improve. There is no special treatment for those who acquire nephrotic syndrome and renal impairment. Aside from meeting the therapeutic needs of this small group of patients, further research is valuable, giving new insights into IgAN pathobiology, which will also improve our understanding of the mechanisms underlying the IgA deposition, thereby leading to new advances in IgAN therapy.

### 3.2. Systemic diseases

Celiac disease and IgAN are related, and a gluten-free diet improves renal involvement clinically and immunologically. Celiac disease and inflammatory bowel diseases are linked with secondary IgAN. A third of Celiac patients have glomerular IgA deposition without any clinical manifestations of renal involvement. Malignant diseases such as a buccal cavity, nasopharynx, and bronchial carcinoma are associated with IgA renal mesangial deposition and proteinuria.

### 3.3. Infectious diseases

Secondary IgAN occurs in viral infections such as human immunodeficiency virus (HIV), cytomegalovirus, hepatitis B, and hepatitis C. Although typical IgAN is rare in the black race, IgAN is equally found in white and black people with HIV infection.^[11]^ Chronic mucosal and other sites infection (streptococcus, staphylococcus), Lyme’s disease, Chlamydia pneumonia, malaria, and schistosomiasis are all associated with secondary IgA mesangial deposition.^[11]^ Clinically, the affected patients may exhibit hematuria, proteinuria, and sometimes renal insufficiency. Histologically, the kidney changes range from mesangial proliferative GN to collapsing glomerulosclerosis with mesangial IgA deposits.

### 3.4. Familial IgA nephropathy

Even though IgAN is mainly a random disease, data suggest that genetic factors play a role. Several examples of familial disease were identified in Italy, and the USA, and an autosomal dominant type have been related to band 6q22-23.^[12]^ In some patients, an increased frequency of specific HLA groupings has also been recorded. A high serum concentration of galactose-deficit-IgA1 (Gd-IgA1) has been reported in almost all IgAN patients and some of their first-degree relatives. It is also reported that Gd-IgA1 levels have a major dominant gene inheritance with an extra-polygenic component. The inheritance of the serum levels Gd-IgA1 has been established in familial and sporadic IgAN patients.^[13]^ There is reported evidence that the aberrant IgA1 glycosylation is a frequently inherited defect, indicating a merging relation between the pathogenesis of Henoch-Schönlein purpura-induced nephritis and IgAN.^[14]^

## 4. IgA1 glycosylation and IgAN pathogenesis

IgA1 and IgA2 are the two subtypes of IgA in humans. IgA1 has O-glycans linked to the heavy chains of the immune globulin at the hinge region (HR). IgA1 O-glycans are present in two forms. The main form is a galactose-β1-3GalNAc disaccharide called T-antigen, and a minor form is known as mono- or di-sialylated form, commonly defined as sialyl-T antigen. O-glycosylation of IgA1 at HR includes several glycosyltransferases, adding a single monosaccharide stepwise to a developing O-glycan chain into the Golgi apparatus. Previously, it was thought that normal serum IgA1 had less or no galactose-deficient O-glycans.^[15]^ Still, it is now believed that even in healthy people, some terminal or sialylated N-acetylgalactosamine is present.^[16]^

Abnormal O-glycosylation is a crucial step in guiding IgA1 immune complex creation and glomerular deposition.^[16,17]^ The evidence suggests that galactose-deficient IgA1 is present in circulating complexes in IgAN patients,^[16]^ and the IgA mesangial deposits are entirely IgA1 subclass attached mainly to the IgG antibody class.^[18]^ Human serum IgA consists primarily of IgA1 with a modest contribution from IgA2. It is 90% monomeric and 10% polymeric, with a small percentage bound in circulating immune complexes. Hepatocytes rapidly catabolize serum IgA1, giving it a short half-life (5 days).^[19]^ Because of their huge size, these complexes are unlikely to enter the perisinusoidal space of the liver through the tiny endothelial fenestrae, impairing the hepatic clearance mechanism of the immune complex from the circulation.^[20]^ The immune complexes can be filtered through the damaged enlarged glomerular fenestrae and precipitate in the mesangial glomerular tissue.^[21]^ This theory is supported by the presence of huge filtered immune complexes, causing more severe glomerular lesions in animals than tiny complexes.

Although the distinctive feature of IgAN is the mesangial IgA1 deposition, there is no evident association between the extent of IgA1 deposition and the grade of glomerular and tubulointerstitial damage. Understanding the mechanisms that cause IgA1 to be deposited in the mesangial space is vital to understanding the pathophysiology of IgAN.^[22]^ IgAN is appeared to be caused by aberrant glycosylation of O-linked glycans in the hinge region of IgA1, resulting in increased circulation of galactose-deficient IgA1 (Gd-IgA1).^[23]^

Most patients have immune system abnormalities at one stage of their illness, such as increased circulating IgA or other humoral or cellular abnormalities. IgA molecules deposited in the glomerular mesangial tissue have been demonstrated to contain the same glycosylation abnormalities.^[18]^ The abnormal glycosylated IgA1 promotes its mesangial deposition through different mechanisms; its synthesis of pathogenic immune complexes or enhances IgA molecular interactions with renal matrix proteins and/or mesangial cell immune receptors. The complement system is typically activated via the alternative and lectin pathways in IgAN rather than the classical pathway, forming complement components that precipitate the pathogenic IgA1-antibody complex as mesangial deposits.^[17]^ There are currently studies investigating the evidence of complement activity that causes glomerular damage in IgAN. Some of these studies have led to an invention of short interfering RNA molecules targeting complement component 5 (C5) formation, which is currently being further investigated.

In summary, the pathogenesis of IgAN is mediated by the formation of antibodies against abnormal galactosylated IgA, producing IgA1 immune complexes. IgAN development requires the assembly of glycan-specific IgG and IgA antibodies that recognize abnormal galactosylated IgA1. The initial event of IgA1 immune complexes being deposited in the mesangium causes a mesangial–podocyte–tubular interaction involving different mediators. The deposited immune complex causes injury to the podocytes and renal tubular epithelial cells, progressively leading to ESRD. ESRD development is primarily due to glomerular and tubulointerstitial damage from chronic progressive inflammation due to immune complex deposition.

## 5. Theories of IgAN pathogenesis

In recent years, clinical and laboratory investigations have produced widely accepted theories that consider the IgAN an autoimmune illness with a complexed multi-step (multi-hits) pathogenic process.^[24]^ In IgAN patients (step I), the IgG and IgA isotype autoantibodies detect circulating Gd-IgA1 (step II). Following this, IgA1–IgG and IgA1–Immune complexes (step III) are generated, which comprise additional materials such as complement system components.^[17]^ The formed immune complexes are shaped and trapped in the glomeruli’s mesangial layer, producing dysregulation of mesangial cells and kidney tissue damage (step IV).^[24]^ A second theory suggests that the oddly glycosylated IgA1 deposits in the mesangium are lanthanide deposits attached firmly to the newly developing autoantibodies, leading to immune complexes synthesis in situ.^[25]^ The immune deposits cause mesangial cells to multiply and produce extracellular matrix, cytokines, and chemokines in excess amounts, damaging the podocytes and causing proteinuria.^[22]^ Activation of the classical complement pathway appears to have a role in the creation and activities of the circulating complexes.^[17]^ In addition, genetic and environmental factors may also influence or control some of the processes in the pathogenesis of IgAN.^[26]^

**Figure 1.**
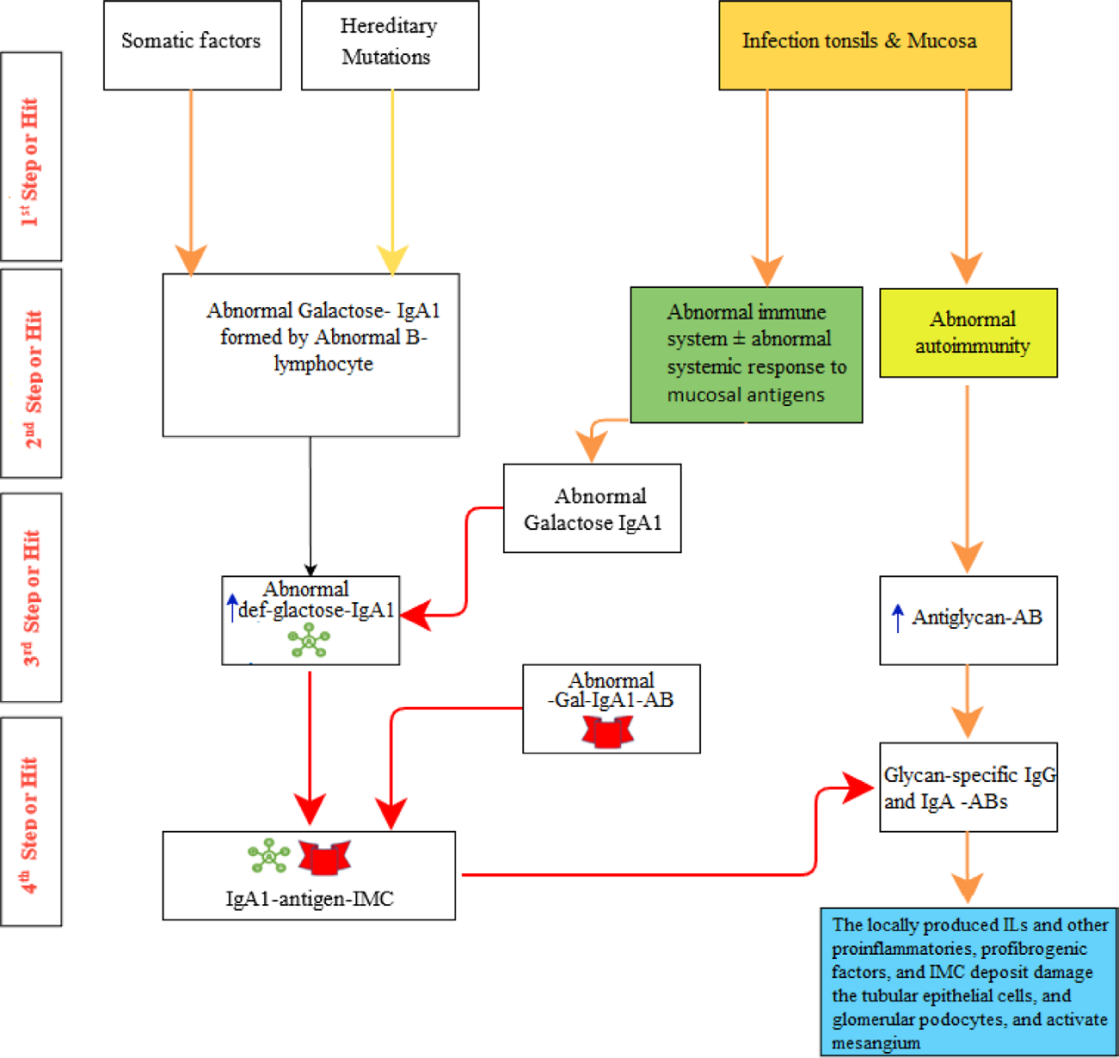
Pathogenesis of IgA nephropathy. Somatic factors and hereditary mutation trigger Abnormal Galactose-IgA1 formation by B-lymphocyte. Infection of mucosa and tonsils stimulates an abnormal immune system ± abnormal systemic response (Hit1). The increased abnormal def-galactose-IgA1 enhances abnormal glycosylated IgA antibody (Abnormal-Gal-IgA1-AB) (Hit 3). Then an immune-mediated complex (IMC) is formed between abnormal def-galactose-IgA1 and Abnormal-Gal-IgA1-AB (IgA1-antigen-IMC), which stimulates the formation of glycan-specific IgG and complex IgA-Antibodies (glycan-specific IgG and IgA-Abs). The glycan-specific IgG and IgA-Abs and locally produced interleukins (ILs) and other proinflammatories, profibrogenic factors, IMC deposit, damage the tubular epithelial cells, glomerular podocytes and mesangial (Hit 4).

Several other reports support the multi-step theory that proposes IgAN pathogenesis. Serum Gd-IgA1 levels may be utilized to predict IgAN progression.^[27]^ The serum levels of Gd-IgA1-specific IgG or IgA autoantibodies are linked with disease severity and may also forecast IgAN progression.^[28]^ Moreover, the blood levels of Gd-IgA1, IgG autoantibodies, soluble CD89 complexes, and IgA1–IgG immune complexes can be used as predictors for disease reappearance in the kidney allograft.^[29]^ Furthermore, the progression of IgAN, according to new clinical and laboratory data results, has driven a paradigm-shifting concept about the autoimmune nature of this condition and the identification of some of the variables linked with genetic diseases.^[26]^ The multi-step theory outlines IgAN’s pathogenetic phases and acts as a “design” for identifying future disease-specific therapeutic targets and generating important biomarkers. The possible pathogenesis mechanisms are summarized in Figure 1.

## 6. Diagnostic approach of IgAN

Thorough urine analysis of a first-void urine sample conducted by an experienced person is the first step in diagnosing IgAN. Red blood cells (RBCs) and RBC cast, which indicate glomerular damage, necessitate a direct study of the urine sediment. Proteinuria testing can be done quantitatively with a 24-hour urine protein measurement or semi-quantitatively with a spot urine protein/creatinine ratio measurement. For adults aged > 50 years with proteinuria, urine protein electrophoresis is usually conducted to rule out monoclonal light chain diseases. Although blood IgA concentration is commonly raised in approximately 50% of IgAN patients, detecting proteinuria in these patients is insensitive and vague and has less clinical significance. Mild proteinuria is relatively common and can occur without microscopic hematuria. Nephrotic-range proteinuria occurs in <5% of IgAN-affected children and adolescents in either early and/or late stages of the disease. Percutaneous kidney biopsy is conducted to confirm the diagnosis of IgAN, particularly in heavy proteinuric IgAN patients. Diffuse proliferative glomerular and minimal-change lesions are the most predominant lesions detected in these patients.

Acute kidney injury (AKI) with edema, oliguria, and hypertension occurs in <5% of IgAN patients. AKI develops mainly due to the following mechanisms: an acute severe immune injury that manifests as necrotizing GN with crescent formation. Secondly, mild glomerular damage is associated with severe hematuria, and kidney impairment is likely due to tubular blockage by RBCs. These clinical presentations can occur in other diseases such as Alport syndrome, Henoch-Schönlein purpura, membranous GN, IgA dominant Post-infectious GN, lupus nephritis, membranoproliferative GN, and thin basement membrane nephropathy. All these diseases present with protein and RBCs urine plus IgA immune complex mesangial deposition. The mild immune injury is generally reversible, and renal function usually improves with suitable supportive measures.

## 7. Treatment

There are contradictory results from several types of research on IgAN therapy. There are currently various treatment options available; however, no universal single therapy regimen is appropriate for all IgAN patients. Supportive treatment, including administering angiotensin-receptor blockers (ARBs) and salt restriction, should be administered to all IgAN patients. A low protein diet is also advisable in nephrotic IgAN patients. Tonsillectomy is indicated for patients who have recurrent chronic tonsillar infections. The MEST-C score’s value (mesangial and endocapillary hypercellularity, segmental sclerosis, interstitial fibrosis/tubular atrophy, and the presence of crescents) in guiding IgAN treatment options have not been proven in randomized controlled trials (RCTs); however, proteinuria appears to be the most relevant predictive feature.

The preferred medications for regulating blood pressure and lowering proteinuria are angiotensin-converting enzyme inhibitors (ACEIs) and angiotensin receptor blockers (ARBs). A study reported that after seven years of follow-up in biopsy-proven cases of IgAN, renal survival in patients with massive proteinuria and normal or moderately impaired renal function was better in the enalapril-treated patients than in the non-enalapril treated patients (92%: 55 % respectively).^[30]^ Another study concluded that combining ramipril with prednisolone was more effective in delaying the progression of kidney damage than ramipril alone in IgAN patients.^[31]^ Some practitioners utilize ACEI and ARB together, but the serum creatinine should be closely monitored. There is not any RCT data supporting this approach.

In IgAN patients with a urinary protein-to-creatinine ratio (UPCR) of <0.5 g/d, there were no clinical trials to weigh the effects of proteinuria reduction on the clinical outcomes. The use of immune-suppressive agents depends mainly upon the progression rate, comorbidities, and histopathological changes of the kidney biopsy. Corticosteroids are currently the cornerstone of IgAN treatment; nonetheless, steroids cannot be used for an extended period due to increased side effects. Although recently there has been a reasonable understanding of the pathogenic mechanisms of IgAN, no IgAN pathogenesis-targeted treatment has been established. Different therapeutic agents such as calcineurin inhibitors (CNIs), cyclophosphamide, mycophenolate mofetil, rituximab, and leflunomide (LEF) are used, but none is approved as a single or combined effective therapy for IgAN.

The main reason for corticosteroid therapy depends upon its anti-inflammatory action, as the pathogenesis of IgAN is predominantly an inflammatory process.^[32]^ Combining intravenous pulse steroid therapy with subsequent oral prednisolone has been shown to reduce protein urine loss and prevent ESRD, increasing the 10-year survival rate.^[33]^ In observational studies and RCTs, the steroid treatment is effective in IgAN patients with proteinuria.^[34]^ Furthermore, the recently discovered role of the gut–kidney axis in IgAN has led to selective corticosteroid formulas targeting the intestinal mucosal immune system, aiming to reduce proteinuria and stabilize kidney function with the least steroid doses.^[35]^ IgAN patients do not continuously improve after steroid therapy. Hence, adding other immunosuppressive agents can accomplish a more substantial synergistic effect. Although IgAN appears as an autoimmune kidney condition, usage of both agents does not reduce protein urine excretion. Furthermore, both agents do not prevent kidney function from gradually decreasing, especially in rapid progressive GN type.^[36]^ The STOP-IgA nephropathy 2008 RCT compared supportive versus immunosuppressive therapy for progressive IgAN reported that combined corticosteroid plus other immune suppressive therapy and supportive care yield better results than supportive care alone.^[37]^

Despite severe infectious complications and higher mortality risk with steroid therapy, their combined use appears promising, especially with other immunosuppressive drugs that act as steroid sparer. Therefore, new well-designed studies are needed to evaluate the best interventions to lower the risks of toxicity and adverse effects of steroids and/or other immune-suppressive drugs in treating IgAN patients.

It is generally recommended that IgAN patients have only hematuria and hypertension; blood pressures, regular urine analysis for RBC, protein, and renal function monitoring are required. However, in proteinuria of <1 g/d, ARBs are the best in the early phases, and blood pressure control should be aggressive, aiming for <130/80 mm Hg.^[38]^ When proteinuria is greater than 1 g/d, the target blood pressure should be <125/75 mm Hg. If a patient excretes >1 g/d, steroids should be used to keep the UPCR within 0.5 to 0.75 g/d.^[39]^ In crescent GN, cyclophosphamide can be tried, but the glomerular filtration rate (GFR) should not be <30 mL/min/1.73 m^2^. However, other experts may prescribe alternative GFR values. Immunosuppressive drug usage is associated with higher rates of drug-related side effects in patients with reduced GFR, but the influence on advanced kidney impairment is uncertain. Immunosuppressive medicines are not advisable when a kidney biopsy indicates significant fibrosis and extensive tubular atrophy. Direct renin inhibitors such as Enalkiren and mineralocorticoid receptor antagonists such as Eplerenone have not been studied in RCTs in IgAN patients. When the GFR is >25 mL/min/1.73 m^2^, and the UPCR is more than 0.6 g/d, a benefit of combined mineralocorticoid receptor antagonists to the other agents has been observed.^[40]^

Tonsillectomy is a common practice in many parts of the world for recurrent severe tonsilitis. Tonsillectomy is conducted in >50% of IgAN Asian patients. Due to the genetic differences in IgA susceptibility and therapeutic responses, the effects of tonsillectomy alone are uncertain.^[41]^ Studies showed that combined pulsed steroid therapy and tonsillectomy improve protein urine loss and the clinical course of IgAN patients, leading to improved patient outcomes.^[42]^ On the contrary, it has also been described that a combined regimen of corticosteroids and tonsillectomy has little or no therapeutic effect on IgAN patients followed up for 20 years, casting doubt about the combination regimen.^[43]^

CNIs have immunosuppressive properties, impairing interleukin-2 (IL-2) generation by weakening T-lymphocyte activation and proliferation, inhibiting the synthesis of secondary cytokines such as IL-4 and tumor necrosis factor. Despite these understood immunomodulatory effects, there is limited clinical-based evidence about the CNIs’ effectiveness in protecting kidney function.^[44]^

Mycophenolate mofetil suppresses T and B lymphocyte proliferation, reducing antibody synthesis, cytotoxic T cell formation, and leukocyte migration to inflammatory areas. However, it conclusively documented that the mycophenolate anti-inflammatory actions did not significantly impair glomerular and interstitial kidney damage in IgAN.^[45]^

Immunomodulatory drugs such as LEF decrease the mitochondrial enzyme dihydroorotate dehydrogenase activity, which is required to synthesize uridine monophosphate for DNA and RNA synthesis. In IgAN, LEF can impair the glomerular autoantibodies and immune complexes mesangial deposits,^[46]^ improving kidney function and urine protein loss.^[46]^ Compared to steroid or ACEI alone or combined with cyclophosphamide, combinations involving LEF were superior and safer. The evidence for the efficacy of LEF is limited, and further studies are needed.

The results of the immune suppression therapy renoprotective trials are inconclusive because they were small, had limited sample sizes, and the trials’ follow-up time was short. A single agent or combined therapy was reported to induce either a complete or partial response; however, some patients may progress to ESRD.^[47]^ Furthermore, ethnicity affects IgAN activity and sensitivity, resulting in global diversity in defining the efficacy of the immune-suppressive drugs and making the meaningful effects of immunosuppressive treatment benefits more difficult to express.^[48]^

## 8. Diet

Although there is no proof, it is advised that IgA kidney deposit patients should avoid meals that enhance antigen exposure of the intestinal mucosa, such as gluten, meat, and milk-based items. It is believed that low-protein diets delay the IgAN progression; however, there are no large trials explicitly designed to study the significant benefit of minimal protein diets in preventing or reducing kidney function in IgAN patients. The Modification of Diet in Renal Disease (MDRD) study was the only significant clinical study that assessed the benefit of a low protein content diet on the deterioration of renal function in CKD patients. Unfortunately, the MDRD study did not specifically test for IgAN patients. Even so, the MDRD study did not show a significant advantage of a low protein diet in impairing kidney function deterioration. Hence, it appears that a low proteins diet cannot be strongly recommended, while it may lead to protein malnutrition, especially if the urine protein loss is high.^[49]^

## 9. Fish oil

Omega-3 is a polyunsaturated fatty acid that acts to decrease leukotrienes and platelet aggregation, reducing IgAN progression. Omega-3 is prescribed to IGAN patients in combination with other drugs. It is given in higher doses, such as 12 g/d, especially for worsened renal function IgAN patients;^[50]^ however, the response is inconsistent.

## 10. Conclusions and prospectives

IgAN is a prevalent disease that does not usually follow a predictable course. IgAN may progress and gradually damages the kidneys. IgAN does not have a uniform heterogenicity worldwide, making it a significant challenge to understand its pathogenesis and develop a strict and specific treatment plan. Hence, further research projects are vital to investigate the exact pathogenetic mechanisms of IgAN.

The protein level in urine appears to be the best clinical guide to follow up on the IgAN progress and evaluate the response to the available treatment. However, renal histology seems to be the most decisive measure to assess the response to the IgAN therapy and the best possible monitoring of the IgAN progression in the long term.

Improving proteinuria and tight blood pressure control by ARBs, ACE, and other supportive measures is essential to impair IgAN progression. Hence, utilization of these measures is not only crucial but is also cost-effective too.

Steroids are the essential treatment agents which appear to reduce proteinuria and the progression of IgAN. However, their long-term adverse effects and the higher dose requirement make their usage questionable. Furthermore, there are conflicting reports about the long-term impact on renal outcomes in IgAN patients. Other immune suppressive and immune-modulating therapies were tried either as a single agent or combined in different regimes. None of the therapeutic regimens effectively treat or prevent IgAN progression. Therefore, more research is needed to explore this area.

Understanding the mechanisms underlying the recurrence of IgAN in the allografts is exciting and needs further assessment, helping to prevent more allografts loss. Kidney transplantation is strongly recommended for IgAN patients with chronic kidney insufficiency, despite the higher recurrence rate. Effectively, kidney transplantation can be conducted more than once, especially if the histological lesion is not crescentic GN type.

Finally, IgAN care is a collaborative effort between the patient, general physicians, and nephrologists. Furthermore, researchers are encouraged to actively study pathophysiology and new therapeutic strategies to prevent additional kidney loss and patient death.

## Author contributions

**Conceptualization:** Elmukhtar Habas.

**Data curation:** Elmukhtar Habas.

**Formal analysis:** Elmukhtar Habas.

**Investigation:** Elmukhtar Habas.

**Methodology:** Elmukhtar Habas, Elrazi Ali, Hafedh Ghazouani.

**Resources:** Elmukhtar Habas, Elrazi Ali.

**Supervision:** Elmukhtar Habas.

**Writing – original draft:** Elmukhtar Habas, Elrazi Ali, Raza Akbar, Abdel-Naser Elzouki.

**Writing – review & editing:** Elmukhtar Habas, Elrazi Ali, Khalifa Farfar, Mahdi Errayes, Jamal Alfitori, Eshrak Habas, Hafedh Ghazouani, Raza Akbar, Fahim Khan, Aisha Al Dab, Abdel-Naser Elzouki.
